# Exploring lifestyle components and associated factors in newly injured individuals with spinal cord injury

**DOI:** 10.1038/s41393-024-01039-9

**Published:** 2024-10-08

**Authors:** Muriel Haldemann, Stevan Stojic, Inge Eriks-Hoogland, Jivko Stoyanov, Margret Hund-Georgiadis, Claudio Perret, Marija Glisic

**Affiliations:** 1 University of Bern, Institute of Social and Preventive Medicine (ISPM), Advanced Study Program Public Health, Bern, Switzerland; 2 Swiss Paraplegic Research, Nottwil, Switzerland; 3 University of Lucerne, Faculty of Health Sciences and Medicine, Lucerne, Switzerland; 4 Institute of Social and Preventive Medicine (ISPM), University of Bern, Bern, Switzerland; 5 REHAB Basel, Basel, Switzerland

**Keywords:** Medical research, Health care

## Abstract

**Study design:**

Cross-sectional analysis from the Inception Cohort of the Swiss Spinal Cord Injury Study (SwiSCI).

**Objectives:**

To describe five lifestyle components in newly injured individuals with spinal cord injury (SCI), explore co-occurrence of these components, and identify associated personal and clinical factors.

**Settings:**

Initial rehabilitation stay following traumatic and non-traumatic SCI.

**Methods:**

Lifestyle components including overweight/obesity, low diet score, physical inactivity, smoking, and alcohol consumption were used independently and to calculate a composite lifestyle score. Analyses were conducted using descriptive statistics, co-occurrence analysis, and multivariate logistic regression.

**Results:**

We included 251 individuals, of whom 77.7% were male, 73.7% suffered from traumatic SCI, and 59.8% had paraplegia. The median age was 51 years (IQR 36–64). Approximately twelve weeks after the injury, more than two-thirds of the study population met the criteria for overweight/obesity, and consumed insufficient amounts of fruits and vegetables, and excessive amounts of meat. Alcohol was consumed by 85.3% of individuals, and 26.8% were current smokers. Almost all study participants met the physical activity guidelines (90 min of moderate to strenuous activity physical activity per week). One-quarter of study participants experienced the co-occurrence of overweight/obesity, low diet score and alcohol consumption. Female sex, younger age and higher education were associated with healthier lifestyle components.

**Conclusion:**

Despite methodological limitations, this study underscores the complexities of healthy lifestyle adherence among individuals newly injured with SCI. It highlights the necessity of improving and implementing screening strategies throughout the continuum of SCI care as early as possible following the trauma.

## Introduction

Increased cardiovascular disease (CVD) risk after spinal cord injury (SCI) is often attributed to a higher prevalence of metabolic syndrome (MetS) that develops as a consequence of a mismatch between daily energy intake and energy expenditure [1]. MetS comprises central obesity, hypertriglyceridemia, low plasma high-density lipoprotein cholesterol (HDL), hypertension, and hyperglycemia [1]. When three or more of the above-mentioned components are present together, they enhance the formation of atherosclerotic plaque and premature CVD development and may represent the same clinical threat as type 2 diabetes (T2D) or an existing coronary artery disease [2]. Physical deconditioning and a hypercaloric diet, although not included in MetS components, together with prolonged oxidative and inflammatory stress, are the major underlying factors that contribute to the development of insulin resistance, hypertension and dyslipidemia following the injury [1, 3, 4].

CVD prevention remains a challenge since the most of predisposing factors are non-modifiable (e.g., sex, ethnicity, and genetic predisposition) [5–7]. Lifestyle interventions, such as continued nutritional guidance, moderate to strenuous intensity exercise and structured behavioral retraining, are among the most important modifiable risk factors and represent the most promising strategies in CVD prevention [8, 9]. Research in SCI population has mainly focused on single lifestyle components and their health benefits in chronic SCI [10–14] and only a limited number of studies addressed lifestyle components in the early injury phase [15–19]. Cardiometabolic disease burden was already high within months post-injury [20–22]. In a recent study, at discharge from initial rehabilitation stay around 40% of study participants met criteria for MetS and around one third were classified as presenting a moderate to high risk for a first CVD event over the next 10 years [20, 21]. Recent guidelines of the Association of the Scientific Medical Societies (AWMF) argue that the initiation of early clinical intervention is central and that patients should already be educated with regards to preventive behaviors during their first rehabilitation [23]. The health benefits of healthy lifestyle go far beyond cardiometabolic disease risk improvement and were linked with improved immune system function, skin, bone and gastrointestinal health, and overall wellbeing [24–28].

In the current study, we aim to describe five major lifestyle components (i.e., overweight/obesity, poor diet, alcohol intake, physical inactivity, and smoking) and explore which of them co-occur in newly injured individuals with SCI. Further, we aim to identify which personal and clinical factors associate with the above-mentioned lifestyle components.

## Methods

### Data source

The Inception Cohort of the Swiss Spinal Cord Injury (SwiSCI) study is a prospective multicenter study recruiting study participants in the four specialized rehabilitation centers in Switzerland (Balgrist University Hospital Zurich, REHAB Basel, Clinique Romande de Réadaptation Sion, and Swiss Paraplegic Center Nottwil) [29]. A wide range of demographic, biopsychosocial, and clinical parameters, as well as biological samples are collected from persons newly diagnosed with traumatic or non-traumatic SCI. The assessments were made at multiple time-points during initial rehabilitation stay (~28 days, ~84 days, ~168 days and around discharge) and one-year post-injury [29]. Participants completing assessments in the Inception Cohort are subsequently followed up in the SwiSCI community survey, which takes place every 5 years. Using May 2020 as a cut-off date, all participants from the Inception Cohort discharged ≥ 2 years ago from the initial rehabilitation were invited to participate in the Community Survey 2022 which is a community-based arm of the SwiSCI study. The current study relies on data collected during the second assessment of the Inception Cohort (approximately 84 days or 12 weeks post-injury, during the initial rehabilitation stay). Unfortunately, due to missing repeated data on lifestyle components longitudinal study was not possible.

### Study population

**Fig. 1:**
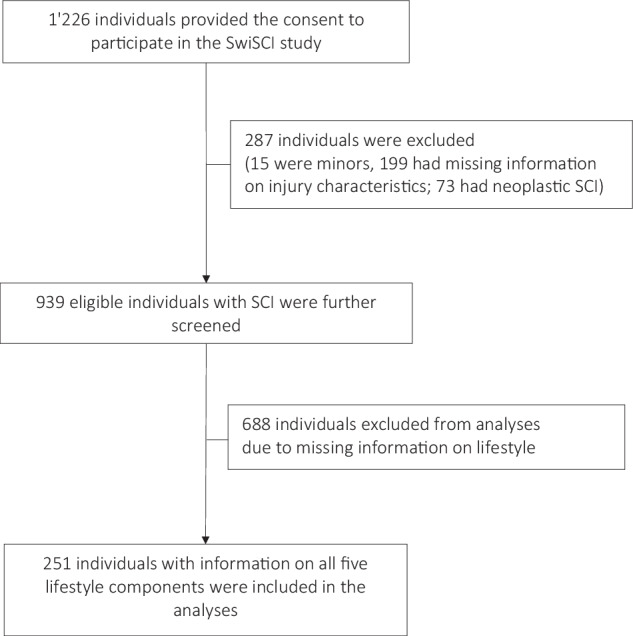
Flow diagram for study participants. The flow diagram shows the selection process of participants included in the study.

The present study considered adult individuals (aged ≥18 years) residing in Switzerland, who were newly diagnosed with traumatic or non-traumatic SCI and had undergone their first inpatient rehabilitation since the start of the SwiSCI study (May 2013) until March 2024. Individuals lacking information on injury characteristics, individuals with neoplastic non-traumatic SCI, and those with missing information on lifestyle components were removed from the study (see Fig. 1).

### Study measures

#### Characteristics of study participants

Clinical and demographic characteristics (i.e., age, sex, years of education) were derived from patients’ medical records. SCI characteristics included SCI etiology (i.e., traumatic versus non-traumatic), causes of injury, and level and completeness of the injury. The level of injury was classified as tetraplegia (level C2–C8) and paraplegia (level T1–S5), and the completeness of injury as motor complete (AIS A and B) and motor incomplete injury (AIS C, D and E), based on the International Standards for Neurological Classification of Spinal Cord Injury (ISNCSCI) [30]. The third version of the Spinal Cord Independence Measure (SCIM III) was used to describe activity limitations [31]. The total score ranges from 0 to 100 with a lower score indicating greater activity limitations and less physical independence.

### Lifestyle components

Waist circumference was measured after bowel care, at the end of normal expiration, approximately between the lower edge of the last palpable rib and the top of the iliac crest. It was measured using a flexible tape measure to an accuracy of 0.5 cm. Weight was measured using an electric scale. The weight of the wheelchair was subtracted from the participant’s total weight with the wheelchair to determine the participant’s weight expressed in kilograms (kg). Both waist circumference and weight were measured once per assessment. Body mass index (BMI) was calculated using the standard formula [weight in kilograms/(height in meters)^2^] [29]. Physical activity was measured using the Physical Activity Scale for Individuals with Physical Disabilities (PASIPD) [32]. To assess dietary habits including fluid intake (L per day, excluding alcoholic beverages), fruit and vegetable intake (portions per day, 1 item on fruit intake, 1 item on vegetable intake: <1, 1–2, 3–4, ≥5), meat intake (portions per week: never, ≤1, 2–3, 4–5, 6, daily) and alcohol intake (frequency per day/week/month and quantity of alcohol intake measured with number of drinks consumed), participants answered questions adopted from the Swiss Health Survey (SHS) [33]. Smoking status, number of years of smoking and number of cigarettes per day were assessed following standardized approach proposed within the ISCoS Pulmonary function basic data set assessments. We classified individuals as with overweight/obesity as a waist circumference ≥ 86.5 cm or BMI ≥ 22 kg/m^2^. Based on the current Swiss nutrition recommendations of the Swiss Nutrition Society, we grouped individuals as adherent or non-adherent to current recommendation for fluid intake, fruit and vegetable intake and meat intake. A nutritional score of 0–4 points was calculated; adherence to a healthy diet is achieved with 3 points or more, while a lower score (0–2 points) is considered as non-adherence to a healthy diet. We used a recent guideline published by Ginis et al. to define the cut off for physical activity which proposes cardiometabolic health with engagement in at least 30 min of moderate to vigorous intensity aerobic exercise 3 times per week [34–38]. Thus, physically active were those individuals reaching at least 90 min per week of moderate or strenuous activity. Individuals who consumed alcohol less than once a month or who never drink were classified as non-drinkers, and the others were classified as consuming alcohol. Smoking refers to study participants who reported actively smoking on the day of interview. The lifestyle score was created as a sum of these five scores providing a lifestyle score ranging from 0 to 5, with higher scores indicating higher adherence to healthy lifestyle habits. Currently, there are no studies linking lifestyle score with SCI outcomes, thus, we used the median total score value to dichotomize study participants into those with high score (scoring 4 or 5 points out of maximum of 5 points) and those with low score (scoring 0–3 points out of maximum of 5 points). Details are provided in **Online Appendix I**.

### Statistical analyses

Descriptive statistics was applied to summarize the baseline characteristics of individuals who had information on all five lifestyle components. To explore the possibility of non-response bias, we compared the baseline characteristics of those who were included in analyses and those for whom the lifestyle score could not be computed by using the chi-squared test or Wilcoxon rank-sum test as applicable. To address the primary aim of our study, we provided a descriptive summary of the five lifestyle components and the overall lifestyle score. To understand a complex interplay between lifestyle components, we provided a graphical summary of poor lifestyle components’ co-occurrence, by applying STATA command “*combination*” and reporting the counts of each unique combination of lifestyle components. To address the secondary aim of our study, we used the equality of proportions test to compare the lifestyle components and lifestyle score between sexes (male vs. female), age groups (elderly or ≥65 years old vs. young), injury etiologies (non-traumatic vs. traumatic), injury severities (tetra- vs. paraplegia) and years of education (≥14 years vs. <14 years). Potential influencing factors were chosen based on the recent systematic review of the literature that explored and summarized the major factors influencing clustering and co-occurrence of multiple risk behaviors [39] and based on availability and completeness of data in the SwiSCI study. For instance, we applied the *“prtest”* command for a two-sample proportion test in STATA which performs a test of the null hypothesis that the proportion of individuals with low nutrition score is the same between the individuals with traumatic vs. non-traumatic SCI. We further used the multivariable logistic regression to explore the association between personal and injury characteristics (i.e., age, sex, injury level, injury etiology, and education) and lifestyle components and overall lifestyle score. All analyses were performed using STATA 17.0 for Windows. A p value lower than 0.05 was considered as statistically significant, but as sensitivity analysis, to account for multiple testing, for each group of results we set an adjusted significance threshold based on the Bonferroni correction such that P for adjustment  < .05 / (number of comparisons). Thus, we adjusted the p value from 0.05 to 0.0017 by applying the Bonferroni correction for the number of tests in regression analysis (n = 30). Further, in the main analyses, we used SCI-adjusted BMI and waist circumference cut-offs to define overweight/obesity. In the sensitivity analyses, we re-ran all analyses using the overweight/obesity definition from the general population. Therefore, overweight/obesity was defined as BMI > 25 kg/m^2^ or waist circumference ≥88 cm for women and ≥102 cm for men [40].

## Results

Among the individuals admitted to first inpatient rehabilitation, 1 226 consented to participate in the SwiSCI study. We removed 287 individuals due to missing information on injury characteristics (n = 199), non-traumatic spinal cord injuries of neoplastic origin (n = 73), and for being underage (n = 15); leaving 939 individuals for further consideration. Overall, 251 individuals had information on all five lifestyle components and formed the study population (see Fig. 1). The median age was 51 years (IQR 36–64), the most of included individuals were male (77.7%) and suffered from traumatic SCI (73.7%). Incomplete motor injury (71.3%) and paraplegia (59.8%) were the most common injury characteristics and the median SCIM III score was 55 points (IQR 35–74). The median duration of rehabilitation was 166 days (IQR 119-208) and median time from injury to rehabilitation admission was 14 days (IQR 8–24), Table 1. When comparing the basic characteristics of study population and individuals with missing information on lifestyle factors, we observed that excluded individuals were older, had higher proportion of non-traumatic SCI, motor incomplete injury and female participants. The proportion of tetra- and paraplegia was similar across included and excluded individuals, and no significant difference was observed in activities limitations (Table 1).

**Table 1:** Characteristics of included study participants.

	Individuals included in current study (N = 251)	Individuals excluded from analyses^a^ (N = 688)	p value for the difference^b^
Age, median (IQR)	51 (36–64)	57 (42–69)	**<0.001**
Sex			
• Male, n (%)	195 (77.7%)	470 (68.3%)	**0.005**
• Female, n (%)	56 (23.3%)	218 (31.7%)	
Education in years, median (IQR)	14 (13–17)	13 (12–16)	**<0.001**
Level of injury			
• Tetraplegia (C2–C8), n (%)	101 (40.2%)	288 (41.9%)	0.65
• Paraplegia (T1–S5), n (%)	150 (59.8%)	400 (58.1%)	
Completeness (AIS Grade)			
• Complete (Grade A & B), n (%)	72 (28.7%)	146 (21.2%)	**0.02**
• Incomplete (Grade C, D & E), n (%)	179 (71.3%)	542 (78.8%)	
SCI etiology			
• Traumatic SCI (TSCI), n (%)	185 (73.7%)	433 (62.9%)	**0.002**
• Non-traumatic SCI (NTSCI), n (%)	66 (26.3%)	255 (37.1%)	
Median time between SCI diagnosis and rehabilitation, days (IQR)	14 (8–24)	14 (9-23)	0.53
Median duration of rehabilitation, days (IQR)	166 (119-208)	130 (74–187)	**<0.001**
SCIM-Score, median (IQR)	55 (35–74)	47.5 (31–69.5)	0.06

^a^Individuals with missing information on lifestyle components.

^b^p value is from chi-squared test or Wilcoxon rank-sum test as applicable.

### Lifestyle components during inpatient stay

**Fig. 2:**
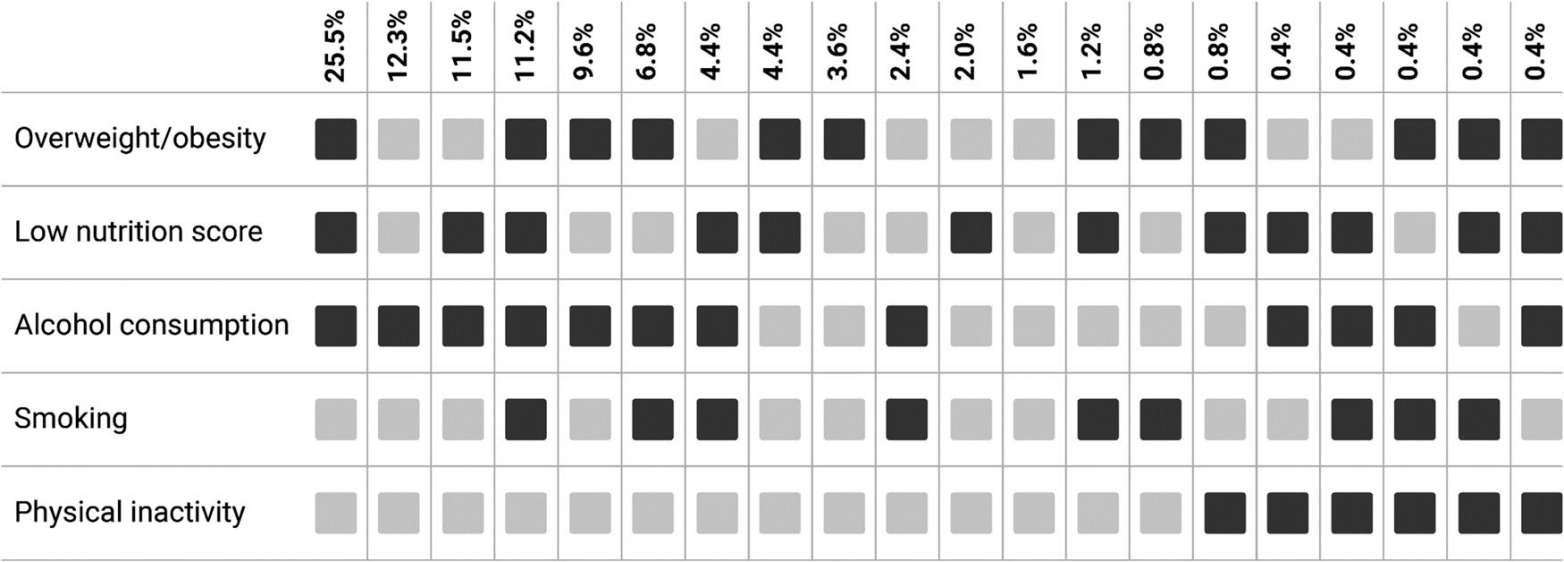
Co-occurrence of poor lifestyle choices. Dark gray squares indicate the presence of an unfavorable lifestyle component or risky behavior, while light gray squares indicate its absence. The numbers denote the percentage of people meeting criteria for the co-occurrence of depicted lifestyle components (i.e., 25.5% of individuals were overweight/obese, have low nutrition score and consume alcohol).

Twelve weeks after the injury, 64.9% of individuals met SCI-specific criteria for overweight/obesity. Among the elderly (≥65 years old) and individuals with non-traumatic SCI, around 80% could be classified as overweight/obese (Tables 2, 3). Alcohol consumption was reported by 85.3% of included individuals, with almost 10% of people consuming alcohol daily. More than two-thirds of included individuals (70.9%) consumed less than five portions of fruit and vegetables a day, and 60.2% consumed more than three meat portions per week. One-quarter of study population (27.9%) were smokers and almost everyone met the recommended 90 min of moderate to strenuous physical activity per week recommendation, Table 2. The Fig. 2 and Supplementary Table 1 show the-cooccurrence of poor lifestyle choices in study population. One quarter of study participant experienced overweight/obesity, low diet score and alcohol consumption co-occurrence (n = 64, 25.5%), whereas low nutrition score (≤2 points) and alcohol consumption were seen in 29 (11.5%) people. The second most common pattern was a one with no obesity, physical activity, and smoking abstinence, yet including alcohol consumption (n = 31, 12.3%). Supplementary Tables 2, 3 show the co-occurrence patterns in men and women. In men, results remain stable, with almost one third experiencing the co-occurrence of overweight/obesity, low diet score and alcohol consumption. The second most common pattern was overweight/obesity, low diet score, alcohol consumption and smoking (12.8%). On the other hand, the most common pattern seen in women was a relatively healthy pattern (including alcohol consumption, no obesity, no smoking, high diet score and physical activity) seen in 23.2% of women, followed by co-occurring pattern of low diet score and alcohol consumption (10.7%).

**Table 2:** The overview of lifestyle components.

Components	Number (%)
**Overweight/obesity (SCI definition)**^**a**^**, n (%)**	163 (64.9%)
**Overweight/obesity (general definition)**^**b**^**, n (%)**	104 (41.43%)
– BMI, kg/m^b^, median (IQR)	24.1 (20.7-27.2)
– Waist circumference, cm, median (IQR)	89 (78.5-99)
**Poor diet score**^**c**^**, n (%)**	157 (62.5%)
– ≤ 1 L of fluid per day	0 (0%)
– <5 portions of vegetables and fruit a day	178 (70.9%)
– >3 portions of meat a week	151 (60.2%)
**Alcohol consumption**^**d**^**, n (%)**	214 (85.3%)
– Less than once a month or never	37 (14.7%)
– 1–3 times a month	59 (23.5%)
– 1–3 times a week	103 (41%)
– 4–6 times a week	28 (11.2%)
– Daily	24 (9.6%)
**Smoking**^**e**^**, n (%)**	70 (27.9%)
– Never smoked	107 (42.6%)
– Ex-smoker	74 (29.5%)
**Physical inactivity**^**f**^**, n (%)**	7 (2.8%)
**Low lifestyle score**^**g**^ **(≤3 points)**	202 (80.5%)

^a^Waist circumference  ≤ 86.5 cm or BMI < 22 kg/m^2^.

^b^Waist circumferences ≥88 cm for women and ≥102 cm for men or BMI > 25 kg/m^2^.

^c^Adherence to a healthy diet is achieved with 3 points or more, while a lower score (0–2 points) is considered as non-adherence to a healthy diet.

^d^Individuals who consume alcohol less than once a month or who never drink as non-drinkers, where everyone else were classified as consuming alcohol.

^e^Smoking refers to study participants who reported actively smoking on the day of interview.

^f^Physical activity refers to engagement in at least 90 min of moderate to vigorous intensity aerobic exercise 3 times per week.

^g^Sum score of five components including overweight/obesity, poor diet, alcohol consumption, smoking and physical inactivity.

### Factors influencing lifestyle components

We observed a higher prevalence of poor diet score (68.2% vs. 42.9%) and alcohol consumption (89.2% vs. 71.4%) in men as compared to women. Women, in general, were more likely to have higher lifestyle score ( ≥ 4 points out of five) points as compared to men (35.7% vs. 14.9%), Table 3. Results from multivariate logistic regression model were in line, and after adjustment for age, injury etiology, injury severity and years of education, women had 4-times higher odds of higher lifestyle score ( ≥ 4 points) as compared to men [OR 4.1 (95% CI 2.0-8.5), p ≤ 0.001]. They were also less likely to consume alcohol [OR 0.3 (95% CI 0.1–0.6), p = 0.002], to report poor diet [OR 0.3 (95% CI 0.2–0.6), p = 0.001], and to have obesity [OR 0.4 (95% CI 0.2–0.6), p = 0.01, the finding was non-significant after Bonferroni correction] Table 4. Elderly (≥65 years old) had higher prevalence of overweight/obesity (82.3%) as compared to younger study population (59.3%), Table 3. When controlling for other factors, the elderly had 2.3-times higher odds of being overweight/obese [(95% CI 1.1–5.0), p = 0.03, non-significant after Bonferroni correction], Table 4. Individuals with non-traumatic injury had higher proportion of overweight/obesity (81.8%) as compared to traumatic SCI (58.9%), and results of logistic regression were in line [the odds of overweight/obesity were 0.4 in traumatic vs. non-traumatic SCI (95% CI 0.2–0.8), p = 0.007, non-significant after Bonferroni correction]. We did not observe statistically significant differences in lifestyle components based on injury level. Finally, the odds of scoring four or five points on overall lifestyle scale, the odds of not consuming alcohol and being non-smoker were lower among people who spent ≥14 years in education vs. the reference group, however, when applying Bonferroni correction, the results did not remain significant, Table 3.

**Table 3:** Lifestyle components comparison based on personal and clinical characteristics.

	Overweight/obesity	p value^a^	Poor Diet	p value^a^	Alcohol Consumption	p value^a^	Physical Inactivity	p value^a^	Smoking	p value ^a^	High lifestyle score (4 & 5)	p value^a^
Male	132 (67.7%)	0.09	133 (68.2%)	0.001^*^	174 (89.2%)	0.001^*^	5 (2.6%)	0.69	54 (27.7%)	0.89	29 (14.9%)	0.0005^*^
Female	31 (55.4%)		24 (42.9%)		40 (71.4%)		2 (3.6%)		16 (28.6%)		20 (35.7%)	
<65 years old	112 (59.3%)	0.001^*^	120 (63.5%)	0.59	164 (86.8%)	0.24	5 (2.6%)	0.81	62 (32.8%)	0.002^*^	39 (20.6%)	0.44
≥65 years old (elderly)	51 (82.3%)		37 (59.7%)		50 (80.76%)		2 (3.2%)		8 (12.9%)		10 (16.1%)	
Non-traumatic SCI	54 (81.8%)	0.001^*^	77 (56.1%)	0.20	55 (83.3%)	0.61	2 (3.0%)	0.89	20 (30.3%)	0.61	10 (15.2%)	0.29
Traumatic SCI	109 (58.9%)		120 (64.9%)		159 (85.9%)		5 (2.7%)		50 (27.0%)		39 (21.1%)	
Tetraplegia	62 (61.4%)	0.33	63 (62.4%)	0.96	84 (83.2%)	0.44	3 (3.3%)	0.89	25 (24.8%)	0.36	24 (23.8%)	0.16
Paraplegia	101 (67.3%)		94 (62.7%)		130 (86.7%)		4 (2.5%)		45 (30%)		25 (16.7%)	
≥14 years of education	95 (59.4%)	**0.01**	99 (61.9%)	0.77	143 (89.4%)	0.01^a^	4 (2.5%)	0.71	39 (24.4%)	0.09	10(11%)	**0.01**
<14 years of education	68 (74.7%)		58 (63.7%)		71 (78.0%)		3 (3.3%)		31 (34.1%)		31 (34.1%)	

^a^p values are from the two-sample proportion test.

^*^Results remain significant after Bonferroni correction (p < 0.0017).

**Table 4:** Association between clinical characteristics of study participants and lifestyle components based on logistic regression analysis.

	Lifestyle components (Dependent variables)											
Independent variables	Overweight/obesity	P value	Poor Diet	P value	Alcohol Consumption	P value	Physical Inactivity	P value	Smoking	P value	High lifestyle score (4 & 5)	P value
	OR (95% CI)		OR (95% CI)		OR (95% CI)		OR (95% CI)		OR (95% CI)		OR (95% CI)	
Male	Ref.	**0.01**	Ref.	**0.001**	Ref.	**0.002**	Ref.	0.70	Ref.	0.99	Ref.	**<0.001**
Female	**0.4 (0.2–0.6)**		0.3 (0.2–0.6)*		0.3 (0.1–0.6)*		1.4 (0.2–7.6)		1.0 (0.5–2.0)		4.1 (2.0-8.5)*	
<65 years old	Ref.	**0.03**	Ref.	0.80	Ref.	0.65	Ref.	0.90	Ref.	**<0.001**	Ref.	0.94
≥65 years old	**2.3 (1.1–5.0)**		0.9 (0.5–1.8)		0.8 (0.3–2.0)		1.1 (0.2–6.8)		0.2 (0.1–0.5)*		1.0 (0.4–2.4)	
Non-traumatic SCI	Ref.	**0.007**	Ref.	0.42	Ref.	0.98	Ref.	0.96	Ref.	0.15	Ref.	0.25
Traumatic SCI	**0.4 (0.2–0.8)**		1.23 (0.7–2.4)		1.0 (0.4–2.3)		0.9 (0.2–5.7)		0.6 (0.3–1.2)		1.6 (0.7–3.8)	
Tetraplegia	Ref.	0.48	Ref.	0.46	Ref.	0.22	Ref.	0.84	Ref.	0.47	Ref.	0.15
Paraplegia	1.3 (0.7–2.1)		1.1 (0.7–2.0)		1.6 (0.7–3.4)		0.9 (0.2–4.0)		1.2 (0.7–2.3)		0.6 (0.3–1.2)	
≥14 years of education	Ref.	0.07	Ref.	0.56	Ref.	0.03	Ref.	0.76	Ref.	**0.02**	Ref.	**0.01**
<14 years of education	0.6 (0.3–1.0)		0.8 (0.5–1.5)		**2.3 (1.1–4.8)**		0.8 (0.2–3.8)		**0.5 (0.3–0.9)**		**2.9 (1.3-6.5)**	

^*^Results remain significant after Bonferroni correction (p < 0.0017)

### Sensitivity analyses

When using the definitions of overweight and obesity from the general population, 41.4% of individuals met the criteria, compared to 64.9% when using the SCI-specific BMI and waist circumference cut-off. Supplementary Tables 4–6 show the co-occurrence of poor lifestyle components using the general population obesity definition. The most common pattern observed was a low nutrition score combined with alcohol consumption, seen in 23.9% of the study population. In contrast, in this analysis, the pattern—obesity, low nutrition score which was the most common when using the SCI specific cut-off for overweight/obesity, and alcohol consumption—dropped to the third position at 13.2%. Elderly individuals, those with non-traumatic SCI, and those with lower education levels had a higher burden of overweight/obesity (see Supplementary Table 7). Results from the logistic regression analysis reflected the same trend, with the elderly having higher odds and those with traumatic SCI having lower odds of overweight/obesity; however, the latter results did not remain significant after Bonferroni correction (see Supplementary Table 8).

## Discussion

A significant proportion of newly injured individuals with SCI had overweight/obesity, particularly the elderly and those with non-traumatic SCI. Alcohol consumption was prevalent among the study participants, with a notable portion consuming alcohol daily. Overweight/obesity, low nutrition scores and alcohol consumption co-occurred in one-quarter of the study population. Meanwhile, only a small fraction of the study population appeared to have a healthy lifestyle pattern while being in a controlled environment, such as inpatient rehabilitation. Sex, age, injury etiology, and education level were associated with lifestyle components, whereas, injury severity appeared not to play a role. Herein, we discuss the most important findings from our study and how they correlate with other evidence in the field.

### Overweight/obesity

Healthy (and unhealthy lifestyle components) co-occur in complex ways that are still insufficiently understood [41]. In our study, overweight/obesity was accompanied by poor dietary choices and alcohol consumption in a large proportion of our study population. In the general population, obesity has been linked to sedentary behavior, physical inactivity, and the consumption of energy-dense food such as sugar-sweetened beverages, alcohol, and snacks as well as a low intake of fruits, vegetables, and whole grains [42]. In addition to those factors, in the SCI population, age at the time of injury, time since injury, injury severity, and hormonal changes (e.g., a decrease in circulating testosterone) further contribute to adverse changes in body composition, beginning within days to weeks post-injury [7, 43–45]. Increasing number of studies have modeled changes in body composition following the injury making these changes foreseeable [46–49]. Structured nutrition and physical activity regimen are largely incorporated in rehabilitation programs, and despite this, percentage of overweight/obesity at discharge from initial rehabilitation remained high [21], thus, appears that currently there is no magic formula to address the complexities of weight control in individuals with SCI [10, 11].

### Diet and alcohol intake

In the context of acute/subacute SCI, studies frequently focused on the risk of malnutrition [50, 51]. This focus is justified given that optimizing macronutrient composition, caloric intake, and nutrient timing, along with the use of selected dietary supplements, can enhance the physiological response to SCI, immobilization and health complications such as skin wounds [52]. Indeed, malnutrition was widespread during inpatient rehabilitation (at three months and prior to discharge), with highest risk being observed in ventilated persons and persons with pressure injuries [51]. In the current study, we reported a discrepancy with current Swiss nutrition guidelines, noting excessive meat intake and insufficient intake of fruits and vegetables during the inpatient stay. Our colleagues assessed the diets among individuals with chronic and acute SCI and did not find a difference between the two groups. Yet, both groups consumed inappropriate amounts of micro- (e.g., very low intakes of vitamins C, D, E, folic acid, pantothenic acid, and biotin) and macronutrients (i.e., insufficient carbohydrates, excess fat) [15]. This is in line with the studies in chronic population that reported excessive energy intake and imbalanced diets comprising too much protein, carbohydrates, and insufficient vitamins and minerals [53]. We reported lower odds of poor diet score in women, which is in line with other studies in general and SCI population, that suggest that women are more likely to maintain healthier diets and are less often overweight/ obese [54]. Similarly, in our study, women were less likely to consume alcohol, which is in line with the findings among the community-dwelling SCI individuals [54]. One fifth of individuals with SCI met the criteria for alcohol abuse or heavy drinking [19, 55–57]. In our study, 41% of individuals consumed alcohol 1–3 times a week, whereas almost 10% of study participants consumed alcohol daily during initial rehabilitation stay. Various factors were associated with alcohol and other psychoactive substances use after the injury, including personality traits, posttraumatic depression, poor coping skills, lack of social support and pain [58]. In early injury phase, we identified male sex, and lower education status as potential factors influencing drinking patterns. These results can help build an effective screening strategy within the rehabilitation program and as a preparation for re-integration into community living.

### Smoking

Smoking prevalence in SCI is largely consistent with the general population, in population-based studies the smoking prevalence was around 35% [13], whereas in clinical cohorts it ranged from 22.6% [59] to 44% [17]. Lower socioeconomic status, binge drinking, and pain medication misuse were associated with smoking [13]. Great proportion of smokers, never tried to quit, and among those who tried, only one third tried to seek professional help [13]. Besides social environment that encourages smoking, concurrent alcohol use has been identified as one of the barriers to smoking cessation [60]. In our study, around one third of individuals were smokers and smoking was mostly co-occurring together with other lifestyle components (e.g., overweight/obesity, low nutrition score and alcohol consumption were seen). This is important when considering development of future preventive strategies and complex interplay between various lifestyle components and health consequences.

### Physical inactivity

Every second person with SCI engages in inactive lifestyle (as compared to approximately 30% in general population) [12, 61–63]. Individuals admitted to rehabilitation undergo personalized exercise regimen, occupational training, and psychological interventions. Thus, a very low proportion of individuals not meeting exercise guidelines seen in our study could be influenced by this structured physical rehabilitation program. We therefore, could not properly explore the association between personal and clinical factors and participation in physical activities. A small study in the Netherlands explored the physical behavior at discharge from initial rehabilitation and at 6- and 12-months post-discharge [16]. On average the mean duration of engagement in physical activity increased and duration of sedentary behavior decreased from discharge to 6 months post-discharge; whereas, in the next 6 month period no significant changes were observed [16]. They reported injury level, age and lower ambulation level as factors influencing physical activity patterns in this transition phase. The main concern remains individuals with high injury levels (above the sixth thoracic segment), who may participate in physical exercise or may not reach the 40–59% of peak oxygen uptake that is considered a minimum to observe health benefits [64], this subpopulation may benefit the most from composite lifestyle and/or behavioral strategies.

### Study limitations and directions for future research

Our study is the first to examine the co-occurrence of five lifestyle components in newly injured individuals with SCI and to explore the association between personal and clinical factors and each lifestyle component, as well as the overall lifestyle score. One-quarter of the study participants experienced a combination of overweight/obesity, low diet scores, and alcohol consumption, indicating that these factors may require special attention during the initial phase of injury recovery. Men, older adults, and individuals with lower educational attainment may struggle to adhere to healthy lifestyle guidelines and could benefit from structured strategies designed to enhance behaviors and establish long-lasting healthy habits.

Although providing important information that can help guide future screening or preventive strategies, our findings should be interpreted with caution. First, the response rate to participation in the SwiSCI study was around 48%, and women, older persons, persons with lower functional independence and those with non-traumatic injury were less likely to participate [29]. Similar findings were observed in our sensitivity analysis when comparing people with complete information on lifestyle components with those with missing information. Thus, our findings may not be generalizable to individuals with more severe injuries, women or elderly. Second, the assessment of lifestyle components is based on self-reported information (i.e., diet, alcohol intake, smoking, and physical activity). While this issue is common in lifestyle research, we cannot exclude the possibility of recall bias (e.g., individuals who follow a special diet or pay close attention to their dietary intake may be more likely to remember and accurately report their habits). Additionally, there is a possibility of social desirability bias, meaning that participants, aware that drinking (while hospitalized) is not desirable, may provide answers they believe will be viewed favorably by others. Also, the diet score has been created based on frequency of fluid, fruits, vegetables and meat intake, and may not provide enough granularity to address the overall diet quality. Further, obesity was defined using clinical assessment of BMI and waist circumference using SCI specific cut-offs, however, since our study measurements were done around 12 weeks post-injury, this cut-off could be overestimating the prevalence of overweight/obesity. Thus, as sensitivity analysis, we applied the general population cut-off for overweight/obesity and our results remain stable. Third, the lifestyle factors were measured around twelve weeks post-injury during initial rehabilitation stay. Since we used the data obtained through a standardized measurement within the SwiSCI study, we did not have information on lifestyle components prior to the injury. The change in health-related behavior often slow and changing for better remains difficult [65], thus, we can argue that some lifestyle habits, such as smoking, dietary or alcohol intake may reflect the habits observed prior to the injury. Changes in body weight and physical activity engagement are more likely to change dramatically after the injury. Perhaps, studies exploring the evolution of lifestyle patterns before and after the injury, could help us understand the behavioral profile of affected individuals and create more effective strategies targeting lifestyle and behavior. For instance, in our analysis age, sex and education were associated with lifestyle components, which is similar as observed in general population. Whereas, injury severity was not associated with lifestyle components, yet, the injury severity, independence in activities of daily living, social support and other environmental factors may be crucial in providing support to develop and maintain healthy lifestyle habits in community setting. Fourth, despite meals being provided by the hospital and the controlled environment designed to promote abstinence from smoking and drinking (without an official ban), our study population still engaged in risky behaviors. We therefore anticipate a potential decline in adherence to a healthy lifestyle during the transition from inpatient rehabilitation to community living, which is a critical period for preventing medical complications such as metabolic syndrome or diabetes [21]. Thus, future studies should explore the lifestyle changes adopted during this transition phase and identify the most important personal and clinical characteristics associated with potential lifestyle components worsening. The complex role of psychosocial factors including social support, needs to be considered alongside other factors. Furthermore, it is necessary to study the association between multiple lifestyle components on health outcomes in both the early injury phase and among those aging with SCI, who may be especially prone to developing health complications (e.g., MetS, CVD, pressure injuries or gastrointestinal problems). Finally, focusing on the synergistic effects of interventions integrating multiple lifestyle components will help develop personalized, sustainable interventions that address multiple risk factors simultaneously. This approach will allow us to identify effective and lasting strategies for disease prevention, improve overall quality of life, and create tailored healthcare approaches for the SCI population.

## Conclusions

This study provides a descriptive summary of five lifestyle components, their co-occurrence, and factors associated with single components and overall lifestyle score in individuals newly diagnosed with SCI and undergoing their first inpatient rehabilitation. Despite the notable methodological limitations of our approach, we offer valuable insights which can be used to address the complexities of SCI care. We identify obesity, alcohol consumption, and diet as factors requiring special consideration during the early injury phase. Men, the elderly, and individuals with lower education levels may particularly struggle to adhere to healthy lifestyle recommendation and may benefit from structured approaches towards improving behavior and building healthy habits in this vulnerable period. Future studies are to replicate our findings in other settings and address methodological issues raised within the current study.

## Supplementary information

Online supplement

## Data Availability

The datasets analyzed during the current study are not publicly available due to the commitment to SwiSCI study participants and their privacy but are available from SwiSCI Study Center (contact@swisci.ch) on reasonable request.
