# Supplementary material for: Exploring lifestyle components and associated factors in newly injured individuals with spinal cord injury
Source: Spinal Cord. 2024 Oct 8;62(12):708–17. doi: 10.1038/s41393-024-01039-9 (PMC11621014; doi:10.1038/s41393-024-01039-9)
Supplement: Supplementary file 1 — Online supplement [file 41393_2024_1039_MOESM1_ESM.docx]

**ONLINE SUPPLEMENT**

**Exploring Lifestyle Components and Associated Factors in Newly Injured Individuals with Spinal Cord Injury**

Muriel Haldemann^1^, Stevan Stojic^2^, Inge Eriks-Hoogland^2,3^, Jivko Stoyanov^2,4^, Margret Hund-Georgiadis^5^,
Claudio Perret^2,3^, Marija Glisic^2,4^

^1^University of Bern, Institute of Social and Preventive Medicine (ISPM), Advanced Study Program Public Health

^2^Swiss Paraplegic Research Nottwil, Switzerland

^3^University of Lucerne, Faculty of Health Sciences and Medicine, Switzerland

^4^Institute of Social and Preventive Medicine (ISPM), University of Bern, Switzerland

^5^REHAB Basel, Switzerland

**Corresponding author:**

PD Dr. M. Glisic, Swiss Paraplegic Research, Guido A. Zäch-Strasse 4, 6207 Nottwil,

+41 41 939 66 45, [marija.glisic@unibe.ch](mailto:marija.glisic@unibe.ch)

**Appendix I:** Classification of lifestyle factors

| **Lifestyle factor** | **Score** | **SCI-specific low risk criteria** | **Assessment tool** |
| --- | --- | --- | --- |
| Overweight/obesity^1^ | 1  0 | Waist circumference (WC) ≤86.5 cm or BMI <22 kg/m^2^  Waist circumference (WC) >86.5 cm or BMI ≥22 kg/m^2^ | Flexible tape measure/ calculation based on weight and height |
| Diet^2^ | 1  0 | Adherence to a healthy diet (Nutrition-Score 3–4 points)  Non-Adherence to a healthy diet (Nutrition-Score 0–2 points) | SwiSCI questionnaire |
| Physical activity^3^ | 1  0 | ≥90 minutes per week of moderate to strenuous activity  <90 minutes per week of moderate to strenuous activity | PASIPD |
| Smoking^4^ | 1  0 | Ex-Smoker and Never-Smoker  Current Smoker | Questionnaire ISCoS Pulmonary |
| Alcohol intake^5^ | 1  0 | Less than once a month or never  More than once a month | SwiSCI questionnaire |
| **Measurement time-point:** 12 weeks after diagnosis, inpatient setting | | | |

^1^Considering the limited accessibility of visceral adipose tissue (VAT) measurement, supine waist circumference (WC) is used, which has been shown to be significantly associated with cross-sectional area (VAT-CSA). Supine WC was chosen because it is less dependent on abdominal muscle tone, which is impaired in individuals with SCI. Using a linear regression model, a supine WC of 86.5cm was chosen as the SCI-specific cutoff point to identify people at risk of developing central adiposity, associated with metabolic imbalance (lipid and carbohydrate profiles). Furthermore, as some experts in the field consider BMI to be a better predictor of central obesity in individuals with SCI, we will consider a person to be centrally obese when meeting either a SCI-specific WC cut-off or a BMI cut-off.^1^

^2^Study participants answered questions on fluid intake (L per day, excluding alcoholic beverages), fruit and vegetable intake (portions per day, 1 item on fruit intake, 1 item on vegetable intake: <1, 1–2, 3–4, ≥5) and meat intake (days/week: never, ≤1, 2–3, 4–5, 6, daily).^2^ Based on available information on nutritional habits and by consulting current Swiss nutrition recommendations of the [Swiss Nutrition Society](https://www.sge-ssn.ch/), we grouped individuals as adherent or non-adherent to healthy diet recommendations. A nutritional score of 0–4 points can be obtained (see **Box I**). Adherence to a healthy diet is achieved with 3 points or more, while a lower score (0–2 points) is considered as non-adherence to a healthy diet.

^3^Physical activity was measured based on the frequency (number per week) and duration (hours per cycle), according to intensity (moderate to strenuous). Physical activity was assessed using the Physical Activity Scale for Individuals with Physical Disabilities (PASIPD). Classification was based on the latest recommendations for physical activity for people with SCI.^2^

^4^Smoking status was assessed using ISCoS Pulmonary function basic data set, number of years of smoking, number of cigarettes per day will be used to classify individuals as smokers, never smokers and ever smokers. Never smokers are defined as participants who reported never having smoked, current smokers as participants who reported actively smoking on the day of interview, and ever smokers as participants who had smoked in the past but did not report actively smoking at the time of the questionnaire.

^5^The frequency and quantity of alcohol intake will be used to classify individuals who consume alcohol less than once a month or who never drink.

**Box I:** Classification of healthy diet (Nutrition-Score)

| Intake of… | Score | Quantity per day or per week |
| --- | --- | --- |
| fluid | 1  0 | ≥1 Liter per day  <1 Liter per day |
| fruit | 1  0 | ≥1 portion per day  <1 portion per day |
| vegetables | 1  0 | ≥3 portions per day  <3 portion per day |
| meat | 1  0 | ≤3 portions per week  >3 portions per week |

**Appendix II**. Co-occurrence of poor lifestyle choices

**Supplemental table 1:** Co-occurrence of poor lifestyle choices in overall population

| **Combination^1^** | **Frequency** | **Percent** |
| --- | --- | --- |
|  |  |  |
| 11100 | 64 | 25.50 |
| 00100 | 31 | 12.35 |
| 01100 | 29 | 11.55 |
| 11110 | 28 | 11.16 |
| 10100 | 24 | 9.56 |
| 10110 | 17 | 6.77 |
| 01110 | 11 | 4.38 |
| 11000 | 11 | 4.38 |
| 10000 | 9 | 3.59 |
| 00110 | 6 | 2.39 |
| 01000 | 5 | 1.99 |
| 00000 | 4 | 1.59 |
| 11010 | 3 | 1.20 |
| 10010 | 2 | 0.80 |
| 11001 | 2 | 0.80 |
| 01101 | 1 | 0.40 |
| 01111 | 1 | 0.40 |
| 10111 | 1 | 0.40 |
| 11011 | 1 | 0.40 |
| 11101 | 1 | 0.40 |
|  |  |  |
| **Total** | **251** | **100.00** |
| **^1^**Lifestyle components are listed as follows: overweight/obesity, Low nutrition Score, Alcohol Consumption, Smoking and Physical inactivity. One depicts poor lifestyle choice, zero depicts good lifestyle choice | | |

**Supplemental table 2.** Co-occurrence of poor lifestyle choices in men

| **Combination^1^** | **Frequency** | **Percent** |
| --- | --- | --- |
|  |  |  |
| 11100 | 60 | 30.77 |
| 11110 | 25 | 12.82 |
| 01100 | 23 | 11.79 |
| 10100 | 19 | 9.74 |
| 00100 | 18 | 9.23 |
| 10110 | 12 | 6.15 |
| 01110 | 9 | 4.62 |
| 11000 | 7 | 3.59 |
| 00110 | 4 | 2.05 |
| 01000 | 4 | 2.05 |
| 10000 | 4 | 2.05 |
| 00000 | 3 | 1.54 |
| 01101 | 1 | 0.51 |
| 01111 | 1 | 0.51 |
| 10010 | 1 | 0.51 |
| 10111 | 1 | 0.51 |
| 11001 | 1 | 0.51 |
| 11010 | 1 | 0.51 |
| 11101 | 1 | 0.51 |
|  |  |  |
| **Total** | **195** | **100.00** |
| **^1^**Lifestyle components are listed as follows: overweight/obesity, Low nutrition Score, Alcohol Consumption, Smoking and Physical inactivity. One depicts poor lifestyle choice, zero depicts good lifestyle choice | | |

**Supplemental table 3.** Co-occurrence of poor lifestyle choices in women

| **Combination^1^** | **Frequency** | **Percent** |
| --- | --- | --- |
|  |  |  |
| 00100 | 13 | 23.21 |
| 01100 | 6 | 10.71 |
| 10000 | 5 | 8.93 |
| 10100 | 5 | 8.93 |
| 10110 | 5 | 8.93 |
| 11000 | 4 | 7.14 |
| 11100 | 4 | 7.14 |
| 11110 | 3 | 5.36 |
| 00110 | 2 | 3.57 |
| 01110 | 2 | 3.57 |
| 11010 | 2 | 3.57 |
| 00000 | 1 | 1.79 |
| 01000 | 1 | 1.79 |
| 10010 | 1 | 1.79 |
| 11001 | 1 | 1.79 |
| 11011 | 1 | 1.79 |
|  |  |  |
| **Total** | **56** | **100.00** |
| ^1^Lifestyle components are listed as follows: overweight/obesity, Low nutrition Score, Alcohol Consumption, Smoking and Physical inactivity. One depicts poor lifestyle choice, zero depicts good lifestyle choice | | |

**Supplemental table 4.** Co-occurrence of poor lifestyle choices in overall population (using general population definition of overweight/obesity)

| **Combination^1^** | **Frequency** | **Percent** |
| --- | --- | --- |
|  |  |  |
| 01100 | 60 | 23.90 |
| 00100 | 40 | 15.94 |
| 11100 | 33 | 13.15 |
| 11110 | 20 | 7.97 |
| 01110 | 19 | 7.57 |
| 10100 | 15 | 5.98 |
| 10110 | 14 | 5.58 |
| 00110 | 9 | 3.59 |
| 01000 | 9 | 3.59 |
| 00000 | 7 | 2.79 |
| 11000 | 7 | 2.79 |
| 10000 | 6 | 2.39 |
| 10010 | 2 | 0.80 |
| 11001 | 2 | 0.80 |
| 11010 | 2 | 0.80 |
| 01010 | 1 | 0.40 |
| 01101 | 1 | 0.40 |
| 01111 | 1 | 0.40 |
| 10111 | 1 | 0.40 |
| 11011 | 1 | 0.40 |
| 11101 | 1 | 0.40 |
| **Total** | **251** | **100.00** |
| ^1^Lifestyle components are listed as follows: overweight/obesity, Low nutrition Score, Alcohol Consumption, Smoking and Physical inactivity. One depicts poor lifestyle choice, zero depicts good lifestyle choice | | |

**Supplemental table 5.** Co-occurrence of poor lifestyle choices in men (using general population definition of overweight/obesity)

| **Combination^1^** | **Frequency** | **Percent** |
| --- | --- | --- |
|  |  |  |
| 01100 | 54 | 51.79 |
| 11100 | 29 | 90.26 |
| 00100 | 27 | 16.92 |
| 11110 | 18 | 100.00 |
| 01110 | 16 | 60.51 |
| 10100 | 10 | 67.18 |
| 10110 | 10 | 72.31 |
| 01000 | 7 | 23.59 |
| 00000 | 6 | 3.08 |
| 00110 | 6 | 20.00 |
| 11000 | 4 | 74.87 |
| 01010 | 1 | 24.10 |
| 01101 | 1 | 52.31 |
| 01111 | 1 | 61.03 |
| 10000 | 1 | 61.54 |
| 10010 | 1 | 62.05 |
| 10111 | 1 | 72.82 |
| 11001 | 1 | 75.38 |
| 11101 | 1 | 90.77 |
| **Total** | **195** | **100.00** |
| ^1^Lifestyle components are listed as follows: overweight/obesity, Low nutrition Score, Alcohol Consumption, Smoking and Physical inactivity. One depicts poor lifestyle choice, zero depicts good lifestyle choice | | |

**Supplemental table 6.** Co-occurrence of poor lifestyle choices in women (using general population definition of overweight/obesity)

| **Combination^1^** | **Frequency** | | | **Percent** |
| --- | --- | --- | --- | --- |
|  |  | | |  |
| 00100 | 13 | | | 23.21 |
| 01100 | 6 | | | 10.71 |
| 10000 | 5 | | | 8.93 |
| 10100 | 5 | | | 8.93 |
| 10110 | 4 | | | 7.14 |
| 11100 | 4 | | | 7.14 |
| 00110 | 3 | | | 5.36 |
| 01110 | 3 | | | 5.36 |
| 11000 | 3 | | | 5.36 |
| 01000 | 2 | | | 3.57 |
| 11010 | 2 | | | 3.57 |
| 11110 | 2 | | | 3.57 |
| 00000 | 1 | | | 1.79 |
| 10010 | 1 | | | 1.79 |
| 11001 | 1 | | | 1.79 |
| 11011 | 1 | | | 1.79 |
|  |  | | |  |
| **Total** | | **56** | **100.00** | |
| ^1^Lifestyle components are listed as follows: overweight/obesity, Low nutrition Score, Alcohol Consumption, Smoking and Physical inactivity. One depicts poor lifestyle choice, zero depicts good lifestyle choice | | | | |

**Supplemental table 7.** Obesity comparison using SCI and general population definition based on personal and clinical characteristics

|  | **Obesity SCI definition** | **p value^1^** | **Obesity  General population definition** | **p value^1^** |
| --- | --- | --- | --- | --- |
| Male | 132 (67.7%) | 0.09 | 76 (38.9%) | 0.14 |
| Female | 31 (55.4%) |  | 28 (50%) |  |
| <65 years old | 112 (59.3%) | **0.001**^a^ | 69 (36.5%) | **0.006**^a^ |
| ≥65 years old | 51 (82.3%) |  | 35 (56.4%) |  |
| Non-traumatic SCI | 54 (81.8%) | **0.001**^a^ | 43 (65.1%) | **<0.001**^a^ |
| Traumatic SCI | 109 (58.9%) |  | 61 (33.0%) |  |
| Tetraplegia | 62 (61.4%) | 0.33 | 37 (63.6%) | 0.21 |
| Paraplegia | 101 (67.3%) |  | 67(44.6%) |  |
| ≥14 years of education | 95 (59.4%) | **0.01**^a^ | 55 (35.0%) | **0.008**^a^ |
| <14 years of education | 68 (74.7%) |  | 49 (52.1%) |  |
| ^1^p-value comes from two-sample proportion test  ^a^Results remained significant after Bonferroni correction | | | | |

**Supplemental table 8**: Association between clinical characteristics of study participants and obesity using SCI and general population definition.

|  | **Obesity (Dependent variable)** | |
| --- | --- | --- |
| **Independent variables** | **SCI definition** | **General population definition** |
|  | **OR (95% CI)** | **OR (95% CI)** |
| Male | Ref. | Ref. |
| Female | 0.4 (0.2-0.6) | 1.29 (0.7-2.5) |
| <65 years old | Ref. | Ref. |
| ≥65 years old | **2.3 (1.1-5.0)*** | 1.42 (0.7-2.7) |
| Non-traumatic SCI | Ref. | Ref. |
| Traumatic SCI | **0.4 (0.2-0.8)^a^** | 0.3 (0.2-0.6) |
| Tetraplegia | Ref. | Ref. |
| Paraplegia | 1.3 (0.7-2.1) | 1.2 (0.7-2.1) |
| ≥14 years of education | Ref. | Ref. |
| <14 years of education | 0.6 (0.3-1.0) | 0.5 (0.3-0.9) |
| ^a^Results remain significant after Bonferroni correction  *Results did not remain significant after Bonferroni correction | | |

**References**

1. Sumrell RM, Nightingale TE, McCauley LS, Gorgey AS. Anthropometric cutoffs and associations with visceral adiposity and metabolic biomarkers after spinal cord injury. *PLoS One* 2018; **13**(8)**:** e0203049.

2. Martin Ginis KA, van der Scheer JW, Latimer-Cheung AE, Barrow A, Bourne C, Carruthers P *et al.* Evidence-based scientific exercise guidelines for adults with spinal cord injury: an update and a new guideline. *Spinal Cord* 2018; **56**(4)**:** 308-321.
